# Studies on the sand fly fauna (Diptera: Psychodidae) in high-transmission areas of cutaneous leishmaniasis in the Republic of Suriname

**DOI:** 10.1186/1756-3305-6-318

**Published:** 2013-11-04

**Authors:** Alida D Kent, Thiago V Dos Santos, Anielkoemar Gangadin, Ashok Samjhawan, Dennis R A Mans, Henk D F H Schallig

**Affiliations:** 1 Department of Parasitology, Faculty of Medical Sciences, Anton de Kom University of Suriname, Kernkampweg 5, Paramaribo, Suriname; 2 Instituto Evandro Chagas/EvandroChagas Institute, Belem, PA, Brazil; 3 National Zoological Collection of Suriname, Anton de Kom University of Suriname, Paramaribo, Suriname; 4 Department of Entomology, Public Health Service, Ministry of Health, Paramaribo, Suriname; 5 Department of Pharmacology, Faculty of Medical Sciences, Anton de Kom University of Suriname, Kernkampweg 5, Paramaribo, Suriname; 6 Koninklijk Instituut voor de Tropen (KIT)/Royal Tropical Institute, KIT Biomedical Research, Parasitology Unit, Amsterdam, The Netherlands

**Keywords:** Sand fly species, *Lutzomyia*, *Leishmania*, Suriname

## Abstract

**Background:**

Sand flies (Diptera: Psychodidae) are the vectors of *Leishmania* parasites, the causative agents of leishmaniasis. Cutaneous leishmaniasis is an increasing public health problem in the Republic of Suriname and is mainly caused by *Leishmania (Vianna) guyanensis*, but *L. (V.) braziliensis*, *L. (L.) amazonensis*, and *L. (V.) naiffi* also infect humans. Transmission occurs predominantly in the forested hinterland of the country. Information regarding the potential vectors of leishmaniasis in Suriname is limited. This study aims to broaden the knowledge about vectors involved in the transmission of cutaneous leishmaniasis in Suriname. For this purpose, sand flies were characterized in various foci of cutaneous leishmaniasis in the country, the districts of Para, Brokopondo, and Sipaliwini.

**Methods:**

Sand flies were collected in areas around mining plots and villages using CDC light traps in the period between February 2011 and March 2013. They were categorized by examination of the spermathecea (females) and the external genitalia (males).

**Results:**

A total of 2,743 sand fly specimens belonging to 34 different species were captured, including four species (*Lutzomyia aragaoi*, *Lu. ayrozai*, *Lu. damascenoi*, and *Lu. sordellii*) that had never before been described for Suriname. Five percent of the catch comprised *Lu. squamiventris sensu lato*, one female of which was positive with *L. (V.) braziliensis* and was captured in a gold mining area in Brokopondo. Other sand fly species found positive for *Leishmania* parasites were *Lu. trichopyga*, *Lu. ininii*, and *Lu. umbratilis*, comprising 32, 8, and 4%, respectively, of the catch. These were captured at gold mining areas in Brokopondo and Sipaliwini, but the *Leishmania* parasites they had ingested could not be identified due to insufficient amounts of DNA.

**Conclusions:**

The sand fly fauna in Suriname is highly diverse and comprises *Lutzomyia* species capable of transmitting *Leishmania* parasites. Four new *Lutzomyia* species have been found, and four species - *Lu. squamiventris (s.l.)*, *Lu. trichopyga*, *Lu. ininii*, and *Lu. umbratilis* - have been found to harbor *Leishmania* parasites. The latter were among the most abundant species captured. These observations may contribute to the understanding of leishmaniasis transmission and the development of control programs in Suriname.

## Background

Leishmaniasis is a parasitic disease caused by protozoan flagellates of the genus *Leishmania*. It is encountered in eighty-eight tropical and sub-tropical countries throughout the world where it mainly affects poor communities, and has a worldwide prevalence of 12 million cases [1]. Transmission is via the bite of infected female sand flies (Diptera: Psychodidae) of the genus *Phlebotomus* in the Old World or the genus *Lutzomyia* in the New World [2]. There are various clinical manifestations of leishmaniasis, the most common of which are the cutaneous and visceral forms. The cutaneous type ranges from single, self-limiting skin ulcers to more disseminated forms with disfiguring scar formation [3]. The visceral type affects internal organs such as spleen, liver, and bone marrow, and patients with this form usually suffer from fever, weight loss, and an enlarged spleen and liver [4,5].

**Figure 1:**
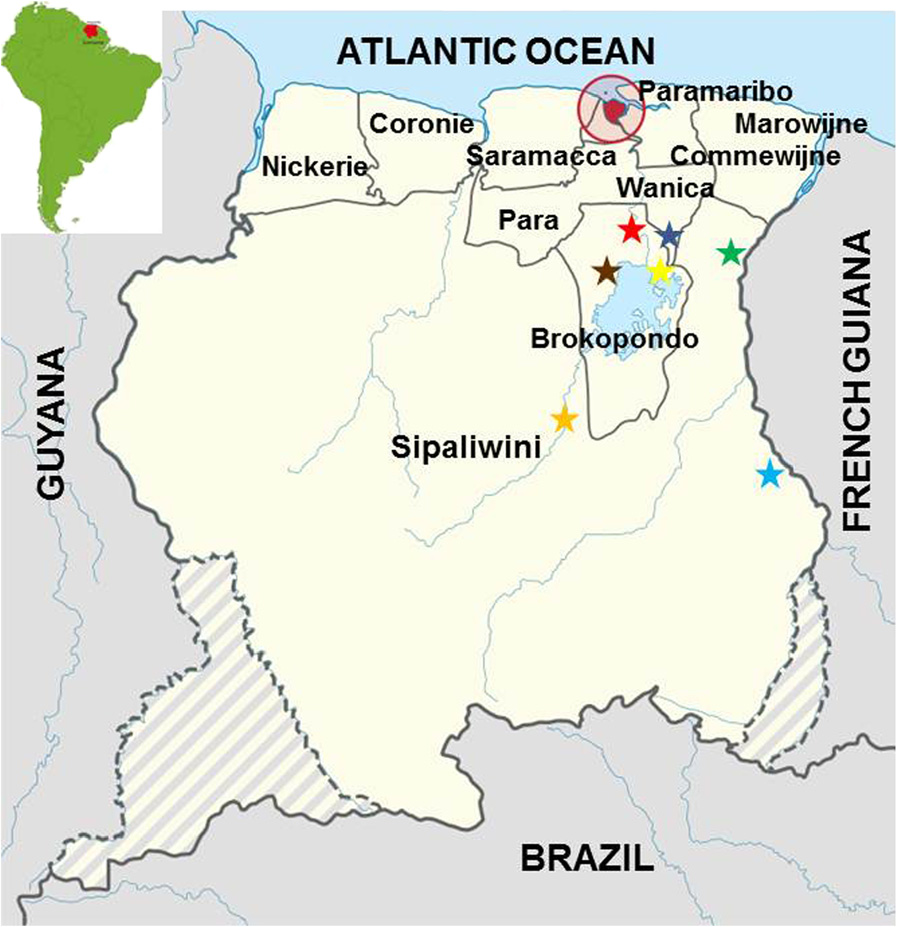
Schematic map of Suriname showing the different districts in the country and sand fly catching sites (“black star symbol” yellow = Brokopondo Centrum; brown = Brownsweg; red = Klaaskreek; purple = Sabajo Hills; green = Merian; light blue = Benzdorp; orange = Pikinslee), and the location of the country on the South American sub-continent (insert).

The Republic of Suriname has a surface area of 163,820 km2, is situated on the north-east coast of South America, and borders the Atlantic Ocean to the north, French Guiana to the east, Brazil to the south, and Guyana to the west (Figure 1). Roughly 80% of the population of about 530,000 lives in the urban-coastal area comprising the capital city of Paramaribo and the Wanica district located in the narrow low-land coastal zone in the northern part of the country (Figure 1). The rural-coastal area of Suriname comprises the districts of Marowijne, Commewijne, Saramacca, Coronie, and Nickerie (Figure 1), and is, together with the southern-rural interior consisting of the districts of Para, Brokopondo, and Sipaliwini (Figure 1), home to the remaining 20% of Suriname’s inhabitants.

The latter part of the country (the hinterland) comprises more than three-quarters of its land surface, and consists largely of sparsely inhabited savanna and dense tropical rain forest. It is mainly populated by Maroons and Amerindians who live in villages along the major rivers. It is also the part of the country with extensive gold mining, bauxite mining, logging, and ecotourism activities that have been growing in scale and economic importance in recent years [6]. Suriname has a tropical climate with abundant rainfall, a uniform temperature of on average 27°C, and a relative humidity of 81% in Paramaribo [7]. There are four seasons, namely the long rainy season (April-July), the long dry season (August-November), the short rainy season (December-January), and the short dry season (February-March).

Cutaneous leishmaniasis is an emerging disease in Suriname where it is known as 'bosyaws’ or 'busiyasi’ [8]. In this country, cutaneous leishmaniasis is endemic, with a zoonotic cycle in which the sand fly vector is in close relationship with the parasites and their wild mammalian reservoir hosts [9]. This cycle takes place primarily in the forested interior of the country where most of the infections occur, and people are most likely to be infected when they intrude in the vectors’ habitat. The Dermatology Service, a division of Suriname’s Ministry of Health that is responsible for the majority of patients with cutaneous leishmaniasis, has reported an increase in the number of cases from 139 to 280 between the years 2006 and 2011 [unpublished data]. This upsurge has been attributed to a higher risk of exposure to the vectors as a result of the above-mentioned increase in economic activities in the hinterland [6].

The majority of cutaneous leishmaniasis cases in Suriname (95.8%) has been attributed to infection with *Leishmania (Vianna) guyanensis*[6]. So far, the sand fly vector that transmits this disease in the country is unknown. A possible candidate is *Lutzomyia umbratilis*[10], the proven vector of *L. (V.) guyanensis* in other countries of the Amazon basin [9,11], and this sand fly species is also present in Suriname [10]. In addition, other *Leishmania* species have recently been identified in Suriname, including *L. (V.) lainsoni* and *L. (L.) amazonensis*[12], *L. (V.) naiffi*[13], as well as *L. (V.) braziliensis*[14]. The sand fly species transmitting these *Leishmania* species have also been identified in other Amazonian countries, but not in Suriname [9,15].

In view of the increasing prevalence of cutaneous leishmaniasis in the country, and considering the ability of *L. (L.) amazonensis* and *L. (V.) braziliensis* to cause the more serious muco-cutaneous form of the disease, it is important to characterize the sand fly species responsible for the transmission of leishmaniasis in Suriname. For this reason, the sand fly diversity in various foci of cutaneous leishmaniasis in the interior of Suriname has been assessed in the present study. The results obtained may improve the understanding of leishmaniasis transmission in the country and contribute to the development of control programs.

## Methods

### Collection sites

Sand flies were collected between February 2011 and March 2013 in the forested hinterland of Suriname. The collection sites were selected on the basis of cases of cutaneous leishmaniasis reported by the Dermatology Service and the Medical Mission. The latter institution is also a division of Suriname’s Ministry of Health and provides primary health care in the hinterland.

The collection sites were grouped according to their locations in the different administrative districts. In the southern district of Sipaliwini (Figure 1), three places were surveyed, the gold mining village of Benzdorp (03˚40′8″N, 54˚5′8″W) in November 2010 and March 2013, the Maroon village Pikinslee (04˚15′17″N, 55˚26′15″W) in April 2012, and the gold mining area of Merian (5˚4′26″N, 54˚29′47″W; 5˚6′16″N, 54˚31′2″W; 5˚5′24″N, 54˚31′59″W) in February 2013. In the centrally located Brokopondo district (Figure 1), gold mining plots in the vicinity of Brokopondo Centrum (05˚1′25″N, 54˚59′33″W) in February 2011, gold mining plots in the vicinity of the Maroon village Klaaskreek (05˚10′50″N, 55˚4′47″W) in April 2011), and Maroon villages around Brownsweg (05˚0′56″N, 55˚10′1″W) were also visited in April 2011. The gold mining area Sabajo Hills (05˚5′38″N, 54˚49′40″W) is situated on the borders of the districts Para, Brokopondo, and Sipaliwini (Figure 1), and was also surveyed in both July 2011and June 2012.

### Sand fly collection and species identification

Sand flies were collected using a total of six CDC light traps. The traps were set for four consecutive nights from 18.00 h to 07.00 h, one to two meters above-ground. In the villages, the traps were placed at peridomestic sites, in dog kennels, chicken coops, and around agricultural plots. In the gold mining locations, the traps were mainly placed in the forest and in animal burrows, far from human residences.

The collected sand flies were sedated by placing them on ice, and males and females were separated. The males were stored in 70% alcohol, then mounted in Berlese’s fluid. The females were stored in Angero NA™ (Mallinckrodt Baker, USA) conservation buffer in preparation for DNA extraction. The sand flies were categorized by species according to the classification system of Young and Duncan [16] by examination of the spermatecea (females) and the external genitalia (males).

### DNA extraction

Five to ten females of the same species were pooled for DNA extraction in 1 mL L6 lysis buffer consisting of 50 mM Tris–HCl (Boehringer, Ingelheim, Ridgefield, CT, USA), 5 M guSCN (Fluka, Buchs, Switzerland), 20 mM EDTA (Merck, Darmstadt, Germany), and 0.1% Triton-X-100 (Packard, Downers Grove, IL, USA), using a protocol described by Boom *et al*. [17]. In brief, the sand flies were disrupted by shaking for 3 min with a 5-mm stainless steel bead in a mini-bead beater-16 model 607 (Biospec Products, Bartlesville, OK, USA). The DNA was trapped by the addition of 30 μL silica gel (Sigma, St. Louis, MO, USA) to the homogenates, mixing for 5 min, and centrifugation for 15 sec at 12,000 × g. The silica pellet was collected, and washed repeatedly with L2 wash buffer (50 mM Tris–HCl (Boehringer, Ingelheim, Ridgefield, CT, USA), 5 M guSCN (Fluka), 70% ethanol, and acetone. Finally, the DNA was eluted in 50 μL TE buffer (Tris EDTA buffer, 100 × concentrated Sigma) and stored at -20°C until analysis.

### Detection of ***Leishmania*** DNA

*Leishmania*-specific DNA was detected by subjecting the DNA samples to quantitative real time PCR (qPCR) according to a protocol described by van der Meide *et al.*[18], with slight modifications. Briefly, each amplification reaction contained 2.5 μL of isolated DNA/RNA sample and was added to 22.5 μL amplification mix containing 1× mastermix (BioRad, Hercules, CA, USA), 0.8 μM each of 18SF and 18SR primer, and 0.2 μM FAM taqman probe. Amplification and real-time measurements were performed in the BioRad opticon minicycler (Biorad, Hercules, CA, USA), under the following conditions: 10 min at 50°C, 5 min at 95°C, followed by 45 cycles of 30 sec at 95°C, and 45 sec at 60°C. The samples were compared to a 10-fold *L. donovani* DNA dilution series ranging from 10 to 10^7^ parasites per reaction.

### Identification of ***Leishmania*** species

The infecting *Leishmania* species were identified using a polymerase chain reaction–restriction fragment length polymorphism (PCR–RFLP) assay described by Marfurt *et al.*[19]. In each PCR run, DNA from *L. (V.) guyanensis* (M4147), *L. (V.) braziliensis*, *L. (L.) amazonensis* (LTB 16), and *L. (V.) naiffi* (L2204) were included as references. The PCR products were incubated for 2 h at 37°C with the restriction enzyme *Hae*III, after which the resulting restriction digestions were analyzed on a 2% agarose (Sphaero Q, Burgos Spain) gel. Fragments were visualized under UV light.

## Results

### Characteristics of collected sand flies according to site of collection

Altogether, 2,743 sand flies were collected in the seven collection sites, including 1,740 males and 1,003 females (Table 1). In the district of Sipaliwini, 1,603 and 244 sand flies were captured in the gold mining areas Merian and Benzdorp, respectively, and 13 in the Maroon village of Pikinslee (Table 1). In the Brokopondo district, 250 and 23 sand flies were collected in the gold mining areas at Klaaskreek and Brokopondo Centrum, respectively, and 29 in Maroon villages at Brownsweg (Table 1). At the gold mining area Sabajo Hills, 581 sand flies were captured (Table 1). Thus, approximately twice as many sand flies were captured in the most inward located district of Sipaliwini (1,830) when compared to the more northern locations in Brokopondo and Sabajo Hills (883). Notably, most of the sand fly captures had been performed during the short dry season (February-March) and the subsequent long rainy season (April-July) (Table 1).

**Table 1:** Sand fly species collected at different foci of cutaneous leishmaniasis in the hinterland of Suriname between February 2011 and March 2013

		**February**		**April**				**July**		**April**		**June**		**February**		**March**		
		**2011**		**2011**				**2011**		**2012**		**2012**		**2013**		**2013**		
		**Brokopondo centrum**		**Klaaskreek**		**Brownsweg**		**Sabajo hills**		**Pikinslee**		**Sabajo hills**		**Merian**		**Benzdorp**		**Total**
	**List of species**	**m**	**f**	**m**	**f**	**M**	**f**	**m**	**f**	**m**	**f**	**m**	**f**	**m**	**f**	**m**	**f**	
1	*Brumptomyia (Brumptomyia) pintoi* (Costa Lima 1932)															1	7	8
2	*Lutzomyia (Aragaoi) aragaoi* (Costa Lima, 1932)	1	1				2	1						33	8			46
3	*Lutzomyia (Ara.) barrettoi barrettoi* (Mangabeira, 1942)								7					7	3	3		20
4	*Lutzomyia (Ara.) inflata* (Floch & Abonnenc, 1944)											1						1
5	*Lutzomyia (Dreisbachi) dreisbachi* (Causey & Damasceno 1945)							2	1									3
6	*Lutzomyia (Evandromyia) infraspinosa* (Mangabeira 1941)	5	4	2			1	2	2			12	3	181	170	5	2	389
7	*Lutzomyia (Eva.) monstruosa* (Floch & Abonnenc 1944)								1						5			6
8	*Lutzomyia (Migonei) migonei* (Franҫa, 1920)														1	1		2
9	*Lutzomyia (Nyssomyia) anduzei* (Rozeboom, 1942)							15	3			1		5	2	6		32
10	*Lutzomyia (Nys.) antunesi* (Coutinho, 1939)		1									2		5		2		10
11	*Lutzomyia (Nys.) flaviscutellata* (Mangabeira, 1942)		1	1			2	2	5			2		18	19			50
12	*Lutzomyia (Nys.) intermedia (Lutz, Neiva, 1912)*								2		1							3
13	*Lutzomyia (Nys.) umbratilis* (Ward & Fraiha, 1977)							2	18		1	6	3	55	28	3	6	122
14	*Lutzomyia (Nys.) whitmani* (Antunes & Coutinho, 1939)				2			4					1	2	2			11
15	*Lutzomyia (Oswaldoi) rorotaensis* (Floch & Abonnenc, 1944)													2	3			5
16	*Lutzomyia (Osw.) trinidadensis* (Newstead, 1922)							19	3				1	9				32
17	*Lutzomyia (Pintomyia) damascenoi* (Mangabeira, 1941)								2					2	7		1	12
18	*Lutzomyia (Psathyromyia) lutziana* (Costa Lima, 1932)															2		2
19	*Lutzomyia (Psa.) punctigeniculata* (Floch & Abonnenc, 1944)															1		1
20	*Lutzomyia (Psa.) shannoni* (Dyar, 1929)							1	2									3
21	*Lutzomyia (Psychodopygus) ayrozai* (Barretto & Coutinho, 1940)	2						1						9	19	3		34
22	*Lutzomyia (Psy) claustrei* (Abonnenc, Léger & Fauran, 1979)			1		2			16				11	11		11	5	57
23	*Lutzomyia (Psy.) corosoniensis* (Le Pont & Pajot, 1978)								1			1	2					4
24	*Lutzomyia (Psy.) davisi* (Root, 1934)			2				7	14	1	2	16	10	10	2	9	12	85
25	*Lutzomyia (Psy.) geniculata* (Mangabeira, 1941)												1			3		4
26	*Lutzomyia (Psy.) hirsuta hirsuta* (Mangabeira, 1942)	1						1	7		1	7	10	4	11	1	12	55
27	*Lutzomyia (Psy.) paraensis* (Cost Lima, 1941)													3				3
28	*Lutzomyia (Psy.) squamiventris sensu lato*							7	43			9	12	37	33		2	143
29	*Lutzomyia (Sciopemyia) fluviatilis* (Floch & Abonnenc, 1944)													2				2
30	*Lutzomyia (Sci.) sordellii* (Shannon & Del Ponte, 1927)												2					2
31	*Lutzomyia (Trichophoromyia) aurensis* (Mangabeira, 1942)					1								1				2
32	*Lutzomyia (Tri.) ininii* (Floch &Abonnenc, 1943)							65	18	2		45	9	26	21	16	16	218
33	*Lutzomyia (Tri.) ubiquitalis* (Mangabeira, 1942)	5	1	223	19	17	4	6				10	5	8		38	16	352
34	*Lutzomyia (Trichopygomyia )trichopyga* (Floch & Abonnenc, 1945)							7	2			9	3	628	185	21	15	870
	not identified	1						7	13	3	2	8	70	4	22	4	20	154
	Total	15	8	229	21	20	9	149	160	6	7	129	143	1062	541	130	114	2743

### Characteristics of collected sand fly species

A total of 154 flies was damaged during capture, mounting, or transportation and could not be identified. The remainder could be categorized in 34 species (Table 1), including 4 that were new for Suriname (46 specimens of *Lu. aragaoi*, 34 of *Lu. ayrozai*, 12 of *Lu. damascenoi*, and 2 of *Lu. sordellii*). The most abundant species were *Lu. trichopyga* (about one-third of the total number of captured flies), as well as *Lu. infraspinosa*, *Lu. ubiquitalis*, and *Lu. innini*, each of which comprised roughly 10% of the total catch (Table 1). About 5% of the collected sand flies could be classified as *Lu. squamiventris sensu lato* and 3% as *Lu. davisi* (Table 1). Approximately 4, 2, and 0.5% of the catch comprised *Lu. umbratilis*, *Lu. flaviscutellata*, and *Lu. whitmani*, sand fly species that had previously been described as possible vectors in Suriname (Table 1).

More than 90% of the 122 sand fly specimens belonging to *Lu. umbratilis* - the proven vector of *L. (V.) guyanensis* – was collected at Merian and Sabajo Hills (Table 1), and almost three-quarters of the 50 specimens belonging to the species *Lu. flaviscutellata* - the proven vector of *L. (L.) amazonensis* - at Merian (Table 1). On the other hand, *Lu. whitmani*, another sand fly species that represents a vector of *L. (V.) guyanensis*, comprised only 0.4% of the total catch of 2,743 specimens (Table 1).

The highest sand fly species diversity was found in the forested locations Sabajo Hills, Merian, and Benzdorp where, 27, 25, and, 20 different species, respectively, were encountered (Table 1). No sand flies were captured near the camps and roads at the gold mining plots of Brokopondo Centrum. The same held true for human residences and stables for domestic animals in or around the villages of Pikinslee and Brownsweg. Only in a dense forested area 1 to 2 meters from a small settlement near Brownsweg, some specimens of *Lu. ubiquitalis* were captured.

### Presence of *Leishmania* DNA

Twenty percent of the female sand flies was analyzed by qPCR. One female specimen of the species *Lu. squamiventris (s.l.)* that was captured at Sabajo Hills and that had visibly taken a blood meal, was positive for *L. (V.) braziliensis* (Table 2). The other sand fly species found positive for *Leishmania* parasites were from pooled samples of *Lu. umbratilis, Lu. ininii, and Lu. trichopyga.* These flies were captured at Sabajo Hills and/or Merian (Table 2), but the *Leishmania* parasites they carried within them could not be identified due to too low or insufficient amounts of DNA.

**Table 2:** ***Lutzomyia *** **species infected with *Leishmamia *** **parasites listed according to sites of collection**

	**Benzdorp**	**Pikinslee**	**Merian**	**Brokopondo centrum**	**Klaaskreek**	**Brownsweg**	**Sabajo hills**
*Lu. squamiventris sensu lato*	-	-	-	-	-	-	*L. (V.) braziliensis*
*Lu. umbratilis*	-	-	*Leishmania* spp.	-	-	-	*Leishmania sp*
*Lu. ininii*	-	-	-	-	-	-	*Leishmania sp*
*Lu. trichopyga*	-	-	-	-	-	-	*Leishmania sp*

## Discussion

Although the *Leishmania* species present in Suriname have been documented, it is still unknown which sand fly vectors are responsible for their transmission. The present study is the first on the sand fly fauna Suriname in almost thirty years, and provides a comprehensive and updated list of sand fly species in different foci of cutaneous leishmaniasis in the country. Four new records for Suriname have been identified (*Lu. aragaoi*, *Lu. ayrozai*, *Lu.sordellii*, and *Lu. damascenoi*), and four sand fly species infected with *Leishmania* parasites are reported (*Lu. squamiventris (s.l.)* with *L. (V.) braziliensis,* and *Lu. umbratilis*, *Lu. ininii*, *Lu trichopyga* with *Leishmania* spp.).

The number of sand fly species collected in this study (34) is close to the number (39) described by Burgos & Hudson [20]. However, the latter number was compiled from collections in thirty different localities, whereas that of the present study was based on numbers encountered in seven foci of cutaneous leishmaniasis in Suriname. These locations were selected in order to increase the likelihood of catching sand flies involved in the transmission of the disease in the country. For the same reason, most surveys were carried out during the months of high and medium rainfall (February through July), when the majority of infections occur (unpublished data, Dermatology Service Paramaribo). These observations may account for the relatively high number of sand fly specimens and species collected in the present study, and the greater numbers captured in forested areas than in villages or camping sites. Notably, infections with *L (V.) guyanensis* - as mentioned above, the main infecting *Leishmania* species in Suriname - typically occur in the hinterland during the rainy seasons [1].

One of the species found for the first time in Suriname was *Lu. ayrozai*. This sand fly is a proven vector of *L. (V.) naiffi* in Brazil [9,21] and of *L. (V.) braziliensis* in Bolivia [15]. *Lu. innini*, *Lu. ubiquitalis*, *Lu. umbratilis*, and *Lu. flaviscutellata* are not new for Suriname. However, *Lu. innini* comprised almost one-tenth of the total catch in the present study, and a French Guianese study reported on specimens of this sand fly species infected with *Leishmania* species [11]. And *Lu. ubiquitalis*, *Lu. umbratilis*, as well as *Lu. flaviscutellata* constituted approximately 10, 4, and 1%, respectively, of the total number of captured sand flies, and are proven vectors of *L. (V.) lainsoni*[21], *L. (V.) guyanensis*[9], and *L. (L.) amazonensis*[9], respectively. Together, these findings are well in agreement with the identification of the various *Leishmania* species as causative agents of leishmaniasis in Suriname [10,12-14], providing a plausible explanation for their presence in the country.

The only *Leishmania* species that could be identified in a sand fly in the present study was *L. (V.) braziliensis*. This parasite was found in a female specimen of *Lu. squamiventris (s.l.)* that is generally considered a vector of *L. (V.) naiffi* in both French Guiana [11] and Brazil [22]. It was captured at Sabajo Hills in the Brokopondo district where possibly one recreational hunter got infected with *L. (V.) braziliensis* (Lai A Fat, personal communication), and two Dutch soldiers with *L. (V.) naiffi*[13]. These data suggest that *Lu. squamiventris (s.l.)* could be a vector of *L (V.) braziliensis* and *L (V.) naiffi* in Suriname, supporting the possible presence of various *Leishmania* species in the country.

Another noteworthy finding from the current study was the presence of *Leishmania* DNA in several female specimens of *Lu. umbratilis*. This observation is in accordance with a previous report [23] mentioning the presence of *Leishmania* promastigotes in the anterior part of the gut of females of this sand fly species. *Lu. umbratilis* is the proven vector of *L. (V.) guyanensis* in French Guiana [24] as well as Brazil and Colombia [15] and a few other areas in the Amazon basin [9,11] including Suriname [10]. Therefore, the detection of *Leishmania* DNA in specimens of this sand fly species in the current study strengthens its candidacy as a vector for this *Leishmania* species in Suriname [10]. However, further studies are needed to definitively incriminate the reported sand fly species as vectors for *Leishmania*. In the present study, only few sandflies were found to carry *Leishmania* parasites (DNA) and more elaborate studies on this topic must be carried out. It is also important to gather information such as infection rates, infectivity, and the presence of metacyclic forms in the mid gut.

## Conclusions

In summary, this study is the first to present a comprehensive and updated list of sand fly species in important foci of cutaneous leishmaniasis in Suriname, including four new records and six proven vectors of the five *Leishmania* species in the country. The abundance and diversity of sand fly species was high in these high-transmission areas, and two sand fly species infected with *Leishmania* parasites (*Lu. squamiventris (s.l.)* with *L. (V.) braziliensis,* and *Lu. umbratilis* with *Leishmania* spp.) have been identified.

The abundance and diversity of sand fly species in Suriname corresponds with the apparent increase in *Leishmania* species in the country. This could be due to more encounters with the vectors as a result of the upsurge in economic activities in the forested hinterland [6]. As shown in the current study, the sand fly vectors are predominantly present in this part of the country. The intensive traveling between Suriname, French Guiana, and Brazil in the gold mining areas may also play an important role in the transmission of leishmaniasis in Suriname [6]. The findings described in the present study contribute to a better understanding of leishmaniasis transmission in Suriname and may aid in the development of programs to control this disease in the country.

## Competing interests

The authors declare that they have no competing interests.

## Authors’ contributions

AK: design of the study, field work, determination of sand flies, molecular analysis, and writing of the manuscript; TS: determination of sand flies, training and advice on catching procedures; AG and AS: catching and determination of sand flies; DM and HS: conception of the study, data analysis, and writing of the manuscript. All authors approved the final version of the manuscript.

## References

[B1] World Health Organization Control of the Leishmaniasis. WHO technical report series 949 2010 Geneva: WHO 21485694

[B2] Killick-Kendrick R The biology and control of phlebotomine sand flies Clin Dermatol 1999 6 279 289 10.1016/S0738-081X(99)00046-2 10384867

[B3] Reithinger R Dujardin JC Louzir H Pirmez C Alexander B Brooker S Cutaneous Leishmaniasis Lancet Inf Dis 2007 6 581 596 10.1016/S1473-3099(07)70209-8 17714672

[B4] Desjeux P Leishmaniasis: current situation and new perspectives Comp Immunol Microbiol Inf Dis 2004 6 305 318 10.1016/j.cimid.2004.03.004 15225981

[B5] Romero GAS Boelaert M Control of Visceral Leishmaniasis in Latin America: a systematic review PLoS NTD 2010 6 1 e584 10.1371/journal.pntd.0000584 PMC2808217 20098726

[B6] van der Meide WF Jensema AJ Akrum RAE Sabajo LOA Lai A Fat RFM Lambregts L Schallig HDFH van der Paardt M Faber WR Epidemiology of cutaneous Leishmaniasis in Suriname: a study performed in Am J Trop Med Hyg 2008 6 192 197 18689623

[B7] Algemeen Bureau voor de Statistiek (ABS) (Paramaribo), Conservation International Suriname Environment Statistics. Issue 286 of Suriname in Cijfers 2012 Paramaribo: Algemeen Bureau voor de Statistiek (ABS)

[B8] Flu PC Die aetiologie der in Surinam vorkommenden sogenannten “Boschyaws” einder der Aleppobeule analogen Erkrankung Centralbl Bakt Parasit Kde I 1911 6 624 637

[B9] Rotureau B Ecology of the Leishmania species in the Guianan ecoregion complex Am J Trop Med Hyg 2006 6 81 96 16407350

[B10] Hudson JE Young DG New records of phlebotomines, leishmaniasis and mosquitoes from Suriname Trans R Soc Trop Med Hyg 1985 6 418 419 10.1016/0035-9203(85)90396-7 2863882

[B11] Fouque F Gaborit P Issaly J Carinci R Gantier JC Racel C Dedet JP Phlebotomine sand flies (Diptera: Psychodidae) associated with changing patterns in the transmission of the human cutaneous Leishmaniasis in French Guiana Mem Ins O Cruz 2007 6 1 35 40 10.1590/S0074-02762007000100005 17293996

[B12] van der Meide WF de Vries HJC Pratlong F van der Wal A Sabajo LOA First reported case of disseminated cutaneous Leishmaniasis caused by *Leishmania (Leishmania) amazonensis* infection, Suriname Emerging Inf Dis 2008 6 5 857 859 10.3201/eid1405.070433

[B13] van Thiel PPMA Gool T van Kager PA Bart A First cases of cutaneous Leishmaniasis caused by *Leishmania (Viannia) naiffi* infection in Suriname Am J Trop Med Hyg 2010 6 588 590 10.4269/ajtmh.2010.09-0360 20348504 PMC2844559

[B14] Hu RVPF Kent AD Adams ER van der Veer C Sabajo LOA Mans DRA de Vries HJC Schallig HDFH Lai A Fat RFM Case report: first case of cutaneous Leishmaniasis caused by *Leishmania (Viannia) braziliensis* in Suriname Am J Trop Med Hyg 2012 6 5 825 827 10.4269/ajtmh.2012.11-0728 22556081 PMC3335687

[B15] Maroli M Feliciangeli MD Bichaud L Charrel RN Grandoni L Phlebotomine sand flies and the spreading of leishmaniasis and other diseases of public health concern Med Vet Entomol 2013 6 123 147 10.1111/j.1365-2915.2012.01034.x 22924419

[B16] Young DG Duncan GA Guide to the identification and geographic distribution of Lutzomyia sand flies in Mexico, the West Indies, Central and South-America (Diptera: Psychodidae) Mem Am Entolomol Inst 1994 6 1 881

[B17] Boom R Sol C Salimans MM Jansen CL Wertheim-van Dillen PM van der Noordaa J Rapid and simple method for purification of nucleic acids J Clin Microbiol 1990 6 495 503 1691208 10.1128/jcm.28.3.495-503.1990 PMC269651

[B18] van der Meide WF Guerra J Schoone GJ Farenhorst M Coelho L Faber WR Peekel I Schallig HDFH Comparison between quantitative nucleic acid sequence based amplification, real-time reverse transcriptase PCR and real-time PCR for quantification of *Leishmania* parasites J Clin Microbiol 2008 6 73 78 10.1128/JCM.01416-07 17959763 PMC2224296

[B19] Marfurt J Nasereddin A Niederwieser I Jaffe C Beck H Felger I Identification and differentiation of *Leishmania* species in clinical samples by PCR amplification of the miniexon sequence and subsequent restriction fragment length polymorphism analysis J Clin Microbiol 2003 6 3147 3153 10.1128/JCM.41.7.3147-3153.2003 12843055 PMC165364

[B20] Burgos AM Hudson JE Annotated list of the Phlebotominae (Diptera) of Suriname Mem Inst O Cruz 1994 6 2 171 178 10.1590/S0074-02761994000200009

[B21] Ferreira-Rangel E Lainson R Proven and putative vectors of American cutaneous Leishmaniasis in Brazil: aspects of their biology and vectorial competence Mem Inst O Cruz 2009 6 7 937 954 10.1590/S0074-02762009000700001 20027458

[B22] Naiff RD Freitas MF Arias JR Barrette TV Momen H Grimaldi JG Epidemiological and nosological aspects of *Leishmania naiffi* Lainson & Shaw 1989 Mem Inst O Cruz 1991 6 317 321 10.1590/S0074-02761991000300006 1842423

[B23] Weijers DJB Linger R Man-biting sand flies in Suriname (Dutch Guiana) *Phlebotomus anduzei* as a possible vector of *Leishmania braziliensis* Annals Trop Parasitol 1966 6 501 508

[B24] Le Pont R Pajot FX Regeur R Preliminary observations on the silvatic cycle of leishmaniasis in French Guiana Trans Roy S Trop Med Hyg 1980 6 1 133 10.1016/0035-9203(80)90032-2 7434410

